# Monitoring for atrial fibrillation prior to patent foramen ovale closure after cryptogenic stroke

**DOI:** 10.1177/17474930221124412

**Published:** 2022-09-19

**Authors:** Hans-Christoph Diener, Rolf Wachter, Andrew Wong, Vincent Thijs, Renate B Schnabel, George Ntaios, Scott Kasner, Peter M Rothwell, Rod Passman, Jeffrey L Saver, Bert A Albers, Richard A Bernstein

**Affiliations:** 1 Department of Neuroepidemiology, Institute for Medical Informatics, Biometry and Epidemiology (IMIBE), Medical Faculty of the University of Duisburg-Essen, Essen, Germany; 2 Department of Cardiology, University Hospital Leipzig, Leipzig, Germany; 3 Neurology Department, Royal Brisbane and Women’s Hospital and the University of Queensland, Brisbane, QLD, Australia; 4 Stroke Theme, The Florey Institute of Neuroscience and Mental Health, Heidelberg, VIC, Australia; 5 Department of Neurology, Austin Health, Heidelberg, VIC, Australia; 6 Department of Cardiology, University Heart & Vascular Center Hamburg, Hamburg, Germany; 7 German Centre for Cardiovascular Research (DZHK), Partner Site Hamburg/Kiel/Lübeck, Hamburg, Germany; 8 Department of Internal Medicine, Faculty of Medicine, School of Health Sciences, University of Thessaly, Larissa, Greece; 9 Department of Neurology, Perelman School of Medicine at the University of Pennsylvania, Philadelphia, PA, USA; 10 Wolfson Centre for Prevention of Stroke and Dementia, Nuffield Department of Clinical Neurosciences, University of Oxford, Oxford, UK; 11 Northwestern University Feinberg School of Medicine, Chicago, IL, USA; 12 Department of Neurology and Comprehensive Stroke Center, David Geffen School of Medicine at UCLA, Los Angeles, CA, USA; 13 Albers Clinical Evidence Consultancy, Winterswijk Woold, The Netherlands; 14 Department of Neurology, Northwestern University Feinberg School of Medicine, Chicago, IL, USA

**Keywords:** Cryptogenic stroke, atrial fibrillation, patent foramen ovale closure, cardiac rhythm monitoring, stroke recurrence, monitoring strategy

## Abstract

**Background:**

Patients who had a cryptogenic stroke (CS) suspected to be causally related to a patent foramen ovale (PFO) are candidates for percutaneous PFO closure. In such patients, it is important to screen for atrial fibrillation (AF). Limited guidance is available regarding AF monitoring strategies in CS patients with PFO addressing optimal monitoring technology and duration.

**Aim:**

To provide a narrative review of cardiac rhythm monitoring in CS patients considered for PFO closure, including current practices, stroke recurrences after CS, findings from monitoring studies in CS patients, and predictors for AF detection published in the literature. To propose a personalized strategy for cardiac monitoring in CS patients, accounting for aspects predicting AF detection.

**Summary of review:**

AF detection in CS patients is predicted by age, left atrial enlargement, prolonged PR interval, frequent premature atrial contractions, interatrial conduction block, diabetes, prior brain infarctions, leukoaraiosis, elevated B-type natriuretic peptide (BNP)/N-terminal pro B-type natriuretic peptide (NT-proBNP) levels, and a family history of AF, as well as composed scores (e.g. CHA_2_DS_2_-VASc, atrial fibrillation in embolic stroke of undetermined source (AF-ESUS)). The causal role of the PFO may be accounted for by the risk of paradoxical embolism (RoPE) score and/or the PFO-Associated Stroke Causal Likelihood (PASCAL) classification.

**Conclusion:**

A personalized approach to AF detection in CS patients is proposed, accounting for the likelihood of AF detection and aimed at obtaining sufficient confidence regarding the absence of AF in patients considered for PFO closure. In addition, the impact of high-risk PFO features on the monitoring strategy is discussed.

## Introduction

Percutaneous closure of patent foramen ovale (PFO) is a safe and effective therapy to prevent recurrent stroke in selected patients 18–60 years of age who had a cryptogenic stroke (CS) with a suspected causal role of a PFO. Randomized trials^1–6^ have reported decreased recurrent stroke rates from percutaneous PFO closure plus medical therapy compared to medical therapy alone, particularly antiplatelet therapy.

Besides the term “cryptogenic stroke,” recent literature often refers to the concept of “embolic stroke of undetermined source” (ESUS). This concept was introduced to have a well-defined diagnostic work-up leading to the diagnosis of ESUS.^7^ ESUS is diagnosed if the stroke is non-lacunar, and no cause of stroke is identified by a standardized diagnostic work-up,^7^ including brain computed tomography or magnetic resonance imaging, 12-lead electrocardiography, precordial echocardiography, cardiac monitoring for ⩾24 h with automated rhythm detection, and imaging of extracranial and intracranial arteries supplying the area of brain ischemia. While CS and ESUS are not interchangeable concepts by definition, it is important to recognize that the great preponderance of patients with CS diagnosed after extensive diagnostic work-up also meet the criteria for ESUS. Most recently, the term PFO-associated stroke was proposed for superficial, large deep, or retinal infarcts in the presence of a medium-risk to high-risk PFO and no other identified likely cause.^8^ Any of these diagnoses cannot be rendered until atrial fibrillation (AF) as a competing cause has been appropriately excluded.

Cardiac rhythm monitoring is typically included in the diagnostic work-up after a stroke to detect paroxysmal AF as a potential cause. Moderate-to-high burden AF is a high-risk source of cardioembolism, indicating guideline-directed chronic oral anticoagulation (OAC).^9^ Clinical evidence does not support PFO closure in this situation. In case of low burden AF, insufficient data are available to indicate if anticoagulation, PFO closure, or both should be pursued. While longer-term monitoring may be utilized to obtain increased confidence about the absence of AF, there is no consistent approach regarding the selection of CS patients considered for PFO closure who are eligible for monitoring and the optimal monitoring technology and duration. A European survey^10^ reported various AF monitoring approaches in CS patients, with most centers (85%) using 24/48 h Holter monitoring and 30% using insertable cardiac monitors (ICMs) in selected patients. This variability is also reflected by current guidelines and consensus statements^11–17^ (see Supplemental Table S1).

Because long-term monitoring in all patients is unlikely to be cost-effective, it is reasonable to tailor the monitoring strategy to the probability of AF detection in various patient groups. The aim of this narrative review is to explore available options for detecting AF and suggest a personalized AF monitoring approach, accounting for the likelihood of detecting AF.

## Methods

As part of this review, a literature search was conducted to identify scientific literature reporting on risk factors or predictors for detection of AF in CS patients. The search strategy is outlined in Table 1.

**Table 1.** Search strategy to identify literature reporting on risk factors/predictors of AF detection in CS patients.

Database	PubMed
Search string	(“cryptogenic stroke” [Title/Abstract] OR “embolic stroke of undetermined source” [Title/Abstract]) AND “atrial fibrillation” [Title/Abstract] AND (“detection” [Title/Abstract] OR “monitoring” [Title/Abstract])
Search period	1 January 2014 to 31 March 2021
Filters	English language
Inclusion criteria	• Reporting on individual risk factors/predictors for detection of AF in patients diagnosed with CS • Risk factors/predictors based on AF detection by means of a systematic ECG monitoring approach utilizing at least one of the following: ○ Long-term ambulatory ECG monitoring ○ External event-triggered recording ○ Mobile Cardiac (Outpatient) Telemetry ○ Insertable Cardiac Monitor • Including ⩾100 patients with initial diagnosis of CS
Exclusion criteria	• Not reporting on individual studies • Reporting on mixed stroke population (CS patients and patients with stroke of known etiology) • Reporting on cardiac rhythm monitoring in other (non-CS) populations or for other purposes than detection of AF

AF: atrial fibrillation; CS: cryptogenic stroke; ECG: electrocardiography.

**Figure 1.**
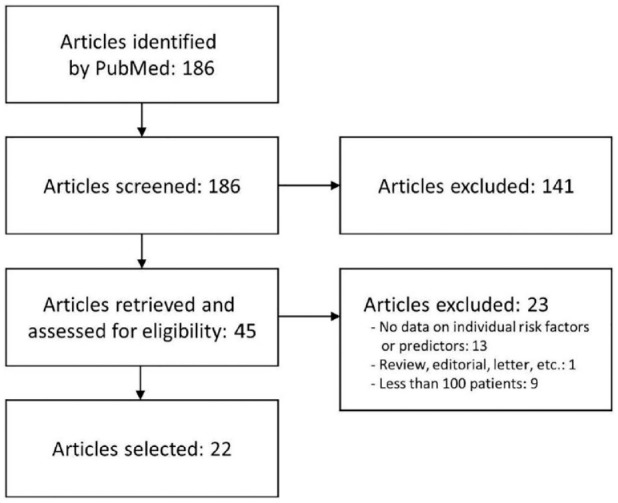
PRISMA flowchart of the literature search for risk factors or predictors of AF detection in CS patients. A number of PRISMA topics did not apply to this narrative review (see text).

The search identified 22 articles reporting on studies utilizing a systematic ECG monitoring approach and reporting on risk factors or predictors for AF detection in a CS population (Figure 1 and Supplemental Figure S1 and Supplemental Table S2). These articles were used as a starting point for this review. While Preferred Reporting Items for Systematic Reviews and Meta-Analyses (PRISMA) principles were followed as much as possible, a number of PRISMA topics were not applied systematically, such as assessments of bias, heterogeneity of study results, confidence in the overall body of evidence, or the evaluation of data in a formal meta-analysis.

The review was conducted by neurologists, internists, and cardiologists with relevant expertise, based on their clinical experience and participation in clinical studies, publications and development of guidelines, or consensus statements related to (cryptogenic) stroke, ECG monitoring, and/or PFO closure.

## AF in CS and recurrent ischemic stroke

Medium-to-high burden AF predisposes to recurrent thromboembolic events. While there is evidence for a weak temporal relationship between AF episodes lasting <24 h and incident stroke,^18,19^ stroke risk was reported to be substantially increased in patients with AF episodes >24 h.^20^ Using monitoring by implantable cardiac rhythm management devices, an atrial tachycardia/AF burden of more than 5.5 h per day appeared to double the risk of thromboembolic events.^21^ The risk of stroke recurrence in the typically young CS patients considered for PFO closure is relatively low. In the control arms of randomized trials on PFO closure, approximately 1.2 strokes per 100 patients per year occurred.^22^ This suggests that deferring PFO closure to pursue prolonged cardiac rhythm monitoring carries a small, but non-zero risk of recurrent stroke during the monitoring interval of approximately 0.1% per month. However, more data are required to reliably determine whether this risk is higher in acute and subacute phases.

## Findings from AF detection studies in CS patients

Several studies have reported on detection of AF in CS patients, generally in those aged 60 years or older (Supplemental Table S3). Application of outcomes from these studies to younger patients typically considered for PFO closure is difficult, given the fact that age is a potent risk factor for AF.

The cryptogenic stroke and underlying AF (CRYSTAL AF) study^23^ randomized 441 CS patients of 40 years or older (mean age = 61.5 years, 22% with a PFO) to ICM monitoring or conventional follow-up. At 6 months follow-up, ICM monitoring detected AF in significantly more patients than conventional follow-up (8.9% vs 1.4%, p < 0.001). In the ICM arm, 75% of first AF episodes were detected within 84 days after randomization. At 3 years, AF was detected in 30% of the patients in the ICM arm, while superiority of ICM monitoring over conventional methods was maintained.

Other studies on ICM monitoring in CS patients have reported AF detection rates ranging from approximately 11% at 6 months to 25% or more after longer monitoring periods. Mostly, patients were older than in the CRYSTAL AF study. Superior AF detection compared to external cardiac monitors or standard care was also reported from studies including patients with known stroke etiology.

Non-invasive long-term monitoring using 30-day external event-triggered loop recording was evaluated in the 30 day event monitoring belt for recording AF after a cerebral ischemic event (EMBRACE) study.^24^ This strategy detected AF in 16.1% of the patients, compared to 3.2% detected by conventional 24 h monitoring. Nevertheless, the post-embolic rhythm detection with implantable vs external monitoring (PER DIEM) trial showed the superiority of ICM monitoring over prolonged non-invasive ECG monitoring,^25^ with AF detected in 15.3% and 4.7% by ICM monitoring and external loop recording, respectively.

The presence of a PFO is associated with a lower AF detection rate. In a meta-analysis^26^ of 14 studies (13,245 patients, mean age 61.2 years), the rate of AF detection was halved in patients with PFO (relative risk (RR) = 0.52, 95% confidence interval (CI) = 0.41–0.63). The rate of AF detection was lower in patients with PFOs found on transthoracic versus transesophageal echocardiography, suggesting that the yield of AF monitoring is further reduced with increasing PFO size. No association between the monitoring method and the likelihood of AF detection was noted.

Overall, studies demonstrated superior AF detection by ICM monitoring compared to non-invasive methods, including shorter-term monitoring and 30-day external event recording. Prolonged monitoring showed that AF is a relatively common finding in mixed age CS patients, with higher yields after longer monitoring periods.

## Predictors for AF detection

**Figure 2.**
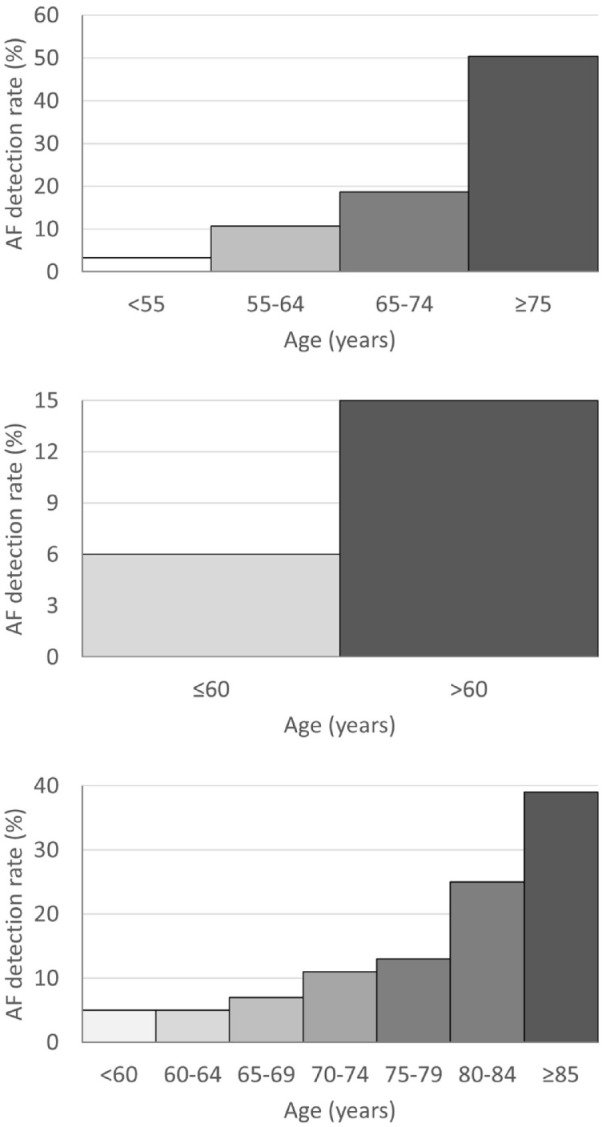
AF detections rates versus age. Top panel: CRYSTAL AF study (1 year ICM monitoring).^27^ Middle panel: Ziegler et al.^30^ (6 months ICM monitoring). Bottom panel: Wachter et al.^31^ (7 days Holter monitoring).

Age is a strong predictor for detecting AF in CS patients (Figure 2). In the CRYSTAL AF study, the likelihood of ICM-detected AF at 12 months roughly doubled with each age decade, with AF rarely detected in patients <55 years.^27^ In other ICM studies, CS patients with no AF detections were younger than those with detections.^28,29^

Similar age-dependent effects were reported from other stroke populations and/or other monitoring methods.^31,32^ Overall, clinical data suggest that AF is an uncommon finding in CS patients <55 years, with the likelihood of detecting AF increasing sharply with older age.

Several other predictors for AF detection in CS patients have been reported. In the CRYSTAL AF cohort, diabetes, prolonged PR interval, and frequent premature atrial contractions were associated with a significantly increased probability of AF detection.^27^ The PR interval was a significant predictor in a multivariate regression model, specifically in patients without PR interval prolonging medication. Frequent premature atrial contractions observed by initial Holter monitoring were also a strong predictor in the EMBRACE study.^33^

Among 196 ESUS patients undergoing 30-day ambulatory heart rhythm monitoring, AF was associated with atrial cardiopathy biomarkers of increased left atrial diameter on echocardiography, P-wave terminal force in electrocardiogram lead V1, and P-R wave interval on electrocardiogram.^34^ Atrial enlargement and interatrial conduction block (maximum P-wave duration ⩾120 ms) were reported as predictors for AF in CS populations monitored by ICM.^35^ Increased levels of B-type natriuretic peptide (BNP) and N-terminal pro B-type natriuretic peptide (NT-proBNP) have also been reported to be associated with AF detection in CS patients using prolonged ECG monitoring, with higher specificity achieved by BNP.^36^

Prior brain infarctions (i.e. not associated with the qualifying index event) and leukoaraiosis were both associated with an almost threefold higher AF detection rate in the CRYSTAL AF study.^37^ Similarly, in CS patients monitored using Mobile Cardiac Outpatient Telemetry (MCOT), prior cortical or cerebellar infarction was an independent predictor of AF.^32^ Given the important role of genetics in the development of lone AF,^38^ a family history of AF may also be considered a predictor for AF detection.

Several composed risk scores are associated with the probability of AF detection (see Supplemental Tables S4 and S5 for risk score descriptions). An increased CHADS_2_ score was associated with an increased AF detection rate in the CRYSTAL AF study.^23^ Similarly, a CHA_2_DS_2_-VASc score ⩾6 was associated with increased AF detection rates.^28^ An AF-ESUS score ⩽0 was reported to have a 100% negative predictive value for the identification of AF episodes lasting >10 h, 83.9% for AF episodes >6 h, and 64.5% for AF episodes >6 min.^39^

The PFO-Associated Stroke Causal Likelihood (PASCAL) Classification System^8^ integrates information regarding the presence of high-risk PFO factors (large shunt size, atrial septal aneurysm (ASA)) and the risk of paradoxical embolism (RoPE) score and provides a categorized likelihood of a causal role of the PFO in stroke pathogenesis. An analysis of pooled individual patient data from six randomized controlled PFO closure trials showed that subsequent AF occurred least often in probable, intermediate in possible, and most often in unlikely PFO-related stroke patients.^40^ This was the case in patients in the control arm, not undergoing PFO closure, as well as in the PFO closure group.

## A personalized approach to cardiac rhythm monitoring in CS patients

**Figure 3.**
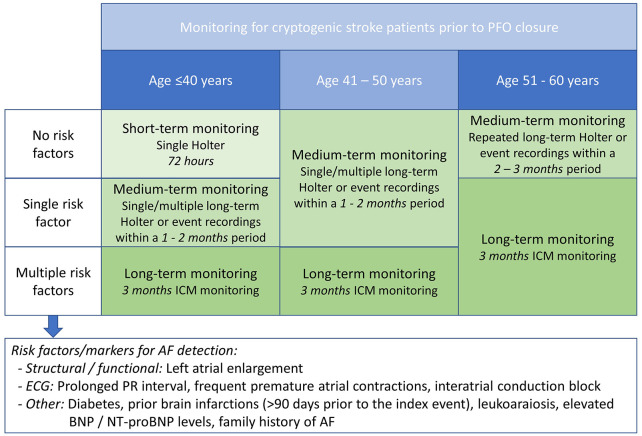
Proposed monitoring approach for exclusion of AF in CS patients considered for PFO closure.

Figure 3 proposes a personalized approach to cardiac rhythm monitoring in CS patients considered for PFO closure. This approach is aimed at providing an acceptable level of confidence that CS patients considered for PFO closure do not have AF. To obtain a similar level of confidence about the absence of AF, low-intensity monitoring is proposed for patients in whom AF is unlikely, while more intensified monitoring is required for patients with a higher likelihood of AF, for example, older patients and those with multiple predictors for AF detection.

Being a strong predictor for AF detection in CS patients, age is used as the primary parameter to select the appropriate monitoring strategy. Further stratification is based on additional “risk factors/markers” identified as predictors for AF detection in studies on long-term rhythm monitoring. Composed risk scores have not been included in this approach in order to avoid duplication and inconsistent weighing of individual risk factors.

Short-term monitoring is considered sufficient for young patients (⩽40 years) with no risk factors predicting AF detection. Consistent with current guidelines for diagnosis and management of AF,^41^ 72-h continuous ECG monitoring is considered sufficient for these patients. More intensified monitoring is suggested for patients aged up to 50 years with a single risk factor/marker for AF detection and for patients aged 51–60 years with no risk factors/markers. Medium-term monitoring may involve long-term Holter monitoring, external long-term event recording, or MCOT. Typically, a single 1-month monitoring period should provide sufficient confidence of absence of AF, but repeated monitoring sessions may provide more confidence. Particularly in patients >50 years of age, repeated monitoring during 2–3 months is suggested. Long-term monitoring is suggested for patients with multiple risk factors/markers (regardless of their age), and for patients aged 51–60 years with ⩾1 risk factors/markers. For these patients, 3-month ICM monitoring is the preferred strategy.^25^

## Discussion

The personalized cardiac rhythm monitoring approach proposed by this review includes monitoring modalities ranging from relatively short-term monitoring in young patients with no risk factors to longer-term monitoring in older patients and patients with predictors for AF detection. While ICMs have demonstrated superior AF detection over other monitoring modalities,^23,25^ their use can be limited to patients with a high risk of having AF. This may partly overcome restrictions regarding the use of the more expensive ICMs due to health-economic considerations and conflicting outcomes regarding the effect of ICM monitoring on the risk of recurrent stroke.^42,43^

It is emphasized that the proposed monitoring approach specifically addresses the objective to exclude AF in patients considered for PFO closure. Cardiac rhythm monitoring may also be performed in other patients to detect the presence of AF, rather than to exclude it, and to inform decisions with regard to antithrombotic therapy. However, this diagnostic setting differs from the setting discussed in this review, and therefore may require different monitoring approaches.

The suggested monitoring approach applies to CS patients up to 60 years of age. While PFO closure in patients ⩾60 years is being considered and evaluated in the literature,^44^ it is currently not supported by randomized clinical evidence. Obviously, PFO closure in these patients should be supported by the most intensive monitoring strategy available.

High-risk PFO features (e.g. large shunt, ASA) may indicate early PFO closure, thereby limiting the duration of pre-closure monitoring. In the CLOSE (Patent Foramen Ovale Closure or Anticoagulants versus Antiplatelet Therapy to Prevent Stroke Recurrence) study,^3^ CS patients with high-risk PFO features undergoing PFO closure without prior long-term ECG monitoring had no recurrent stroke during a mean follow-up period of 5 years. A meta-analysis of PFO closure trials showed a lower likelihood of AF detection in patients with high-risk PFO features and a greater benefit from PFO closure, compared to those without a high-risk PFO.^40^ Therefore, monitoring duration may be fine-tuned based on the presence of high-risk PFO features.

CS patients with a PFO that is a likely cause of the stroke are a distinct subgroup of the ESUS population. An extensive diagnostic work-up is required to precisely delineate this subgroup in order to offer PFO closure to those patients who have a demonstrated benefit of this therapy. In an overall population of ESUS patients, the new approach rivaroxaban inhibition of factor Xa in a global trial vs ASA to prevent embolism in ESUS (NAVIGATE ESUS)^45^ and randomized double-blind evaluation in secondary stroke prevention comparing the efficacy and safety of the oral thrombin inhibitor dabigatran etexilate versus acetylsalicylic acid in patients with ESUS (RE-SPECT ESUS)^46^ randomized trials were not able to demonstrate superiority of dabigatran or rivaroxaban over aspirin in the prevention of stroke recurrence. This may be, at least, partially due to the inhomogeneous nature of the enrolled patient population. A small proportion of patients in these trials had a PFO, and medium- to long-term cardiac rhythm monitoring was only performed in a minority of the patients. Identification of more homogeneous subgroups of ESUS patients related to (probable) causes may be needed to make a well-informed choice between available treatment options, including PFO closure and a variety of pharmacological therapies. Other studies are currently investigating whether the biomarker-indicated degree of atrial cardiopathy or cardiac thrombogenicity may help define subgroups of ESUS patients who specifically benefit from direct oral anticoagulants over antiplatelets (AtRial Cardiopathy and Antithrombotic Drugs In Prevention After Cryptogenic Stroke (ARCADIA), ClinicalTrials.gov Identifier: NCT03192215) or cardiac thrombogenicity (MidregiOnal Proatrial Natriuretic Peptide to Guide SEcondary Stroke Prevention (MOSES), ClinicalTrials.gov Identifier: NCT03961334).

Additional data on the prevalence of AF in CS patients, stratified according to age and other predictors of AF detection, would allow further optimization of risk-based monitoring strategies. In this context, the Catch-up-ESUS study,^47^ initiated in 2018, may provide additional clinical data (ClinicalTrials.gov identifier: NCT03820375). One of the objectives of this study is to determine the rates of ICM-detected AF among various risk strata of patients diagnosed with ESUS.

An additional topic for further research is the benefit and cost-effectiveness of monitoring following PFO closure. Particularly, for patients whose strokes are classified as “Possibly” related to PFO, closure is often indicated but also increases the risk of subsequent clinically apparent AF.^40^ It is important to carefully consider the benefit–risk profile of these patients, accounting for the expected benefits of PFO closure, as well as the need of anticoagulation after PFO closure. In case of PFO closure in these patients, continued monitoring, especially ICM monitoring, may detect AF occurring beyond the peri-procedural period that merits consideration for anticoagulation.

In summary, the monitoring approach proposed by this review should provide the desired level of confidence and justification for PFO closure across all risk strata of CS patients while minimizing treatment delays in patients at low risk of having AF. The use of the proposed monitoring approach may result in an efficient and strategic utilization of cardiac rhythm monitoring resources. This may avoid unnecessary long-term monitoring and promote early PFO closure in younger patients with a low likelihood of having AF, as well as avoid PFO closure in older patients before a reasonable confirmation of the absence of AF has been obtained.

## Supplemental Material

sj-docx-1-wso-10.1177_17474930221124412 – Supplemental material for Monitoring for atrial fibrillation prior to patent foramen ovale closure after cryptogenic stroke Click here for additional data file. Supplemental material, sj-docx-1-wso-10.1177_17474930221124412 for Monitoring for atrial fibrillation prior to patent foramen ovale closure after cryptogenic stroke by Hans-Christoph Diener, Rolf Wachter, Andrew Wong, Vincent Thijs, Renate B Schnabel, George Ntaios, Scott Kasner, Peter M Rothwell, Rod Passman, Jeffrey L Saver, Bert A Albers and Richard A Bernstein in International Journal of Stroke

## References

[1] Sondergaard L Kasner SE Rhodes JF , et al Patent foramen ovale closure or antiplatelet therapy for cryptogenic stroke. N Engl J Med 2017; 377: 1033–1042. 28902580 10.1056/NEJMoa1707404

[2] Saver JL Carroll JD Thaler DE , et al Long-term outcomes of patent foramen ovale closure or medical therapy after stroke. N Engl J Med 2017; 377: 1022–1032. 28902590 10.1056/NEJMoa1610057

[3] Mas JL Derumeaux G Guillon B , et al Patent foramen ovale closure or anticoagulation vs. antiplatelets after stroke. N Engl J Med 2017; 377: 1011–1021. 28902593 10.1056/NEJMoa1705915

[4] Lee PH Song JK Kim JS , et al Cryptogenic stroke and high-risk patent foramen ovale: the DEFENSE-PFO trial. J Am Coll Cardiol 2018; 71: 2335–2342. 29544871 10.1016/j.jacc.2018.02.046

[5] Furlan AJ Reisman M Massaro J , et al Closure or medical therapy for cryptogenic stroke with patent foramen ovale. N Engl J Med 2012; 366: 991–999. 22417252 10.1056/NEJMoa1009639

[6] Meier B Kalesan B Mattle HP , et al Percutaneous closure of patent foramen ovale in cryptogenic embolism. N Engl J Med 2013; 368: 1083–1091. 23514285 10.1056/NEJMoa1211716

[7] Hart RG Diener HC Coutts SB , et al Embolic strokes of undetermined source: the case for a new clinical construct. Lancet Neurol 2014; 13: 429–438. 24646875 10.1016/S1474-4422(13)70310-7

[8] Elgendy AY Saver JL Amin Z , et al Proposal for updated nomenclature and classification of potential causative mechanism in patent foramen ovale-associated stroke. JAMA Neurol 2020; 77: 878–886. 32282016 10.1001/jamaneurol.2020.0458

[9] Steffel J Collins R Antz M , et al 2021 European Heart Rhythm Association practical guide on the use of non-vitamin K antagonist oral anticoagulants in patients with atrial fibrillation. Europace 2021; 23: 1612–1676. 33895845 10.1093/europace/euab065 PMC11636576

[10] D’Andrea A Dweck MR Holte E , et al EACVI survey on the management of patients with patent foramen ovale and cryptogenic stroke. Eur Heart J Cardiovasc Imaging 2021; 22: 135–141. 33346351 10.1093/ehjci/jeaa318 PMC7822641

[11] Horlick E Kavinsky CJ Amin Z , et al SCAI expert consensus statement on operator and institutional requirements for PFO closure for secondary prevention of paradoxical embolic stroke: the American Academy of Neurology affirms the value of this statement as an educational tool for neurologists. Catheter Cardiovasc Interv 2019; 93: 859–874. 30896894 10.1002/ccd.28111

[12] Rubiera M Aires A Antonenko K , et al European Stroke Organisation (ESO) guideline on screening for subclinical atrial fibrillation after stroke or transient ischaemic attack of undetermined origin. Eur Stroke J. 2022; 7: CVII–CXXXIX. 10.1177/23969873221099478 PMC9446336 36082257

[13] Pristipino C Sievert H D’Ascenzo F , et al European position paper on the management of patients with patent foramen ovale. General approach and left circulation thromboembolism. EuroIntervention 2019; 14: 1389–1402. 30141306 10.4244/EIJ-D-18-00622

[14] Wein T Lindsay MP Côté R , et al Canadian stroke best practice recommendations: secondary prevention of stroke, sixth edition practice guidelines, update 2017. Int J Stroke 2018; 13: 420–443. 29171361 10.1177/1747493017743062

[15] Australian clinical guidelines for stroke management—chapter 4 of 8: secondary prevention, https://app.magicapp.org/#/guideline/8L0RME/section/EgV9pn (2021, accessed 17 August 2021).

[16] Kleindorfer DO Towfighi A Chaturvedi S , et al 2021 guideline for the prevention of stroke in patients with stroke and transient ischemic attack: a guideline from the American Heart Association/American Stroke Association. Stroke 2021; 52: e364–e467. 10.1161/STR.0000000000000375 34024117

[17] Messé SR Gronseth GS Kent DM , et al Practice advisory update summary: patent foramen ovale and secondary stroke prevention: report of the guideline subcommittee of the American Academy of Neurology. Neurology 2020; 94: 876–885. 32350058 10.1212/WNL.0000000000009443 PMC7526671

[18] Singer DE Ziegler PD Koehler JL Sarkar S Passman RS . Temporal association between episodes of atrial fibrillation and risk of ischemic stroke. JAMA Cardiol 2021; 6: 1364–1369. 34586356 10.1001/jamacardio.2021.3702 PMC8482300

[19] Brambatti M Connolly SJ Gold MR , et al Temporal relationship between subclinical atrial fibrillation and embolic events. Circulation 2014; 129: 2094–2099. 24633881 10.1161/CIRCULATIONAHA.113.007825

[20] Van Gelder IC Healey JS Crijns H , et al Duration of device-detected subclinical atrial fibrillation and occurrence of stroke in ASSERT. Eur Heart J 2017; 38: 1339–1344. 28329139 10.1093/eurheartj/ehx042

[21] Glotzer TV Daoud EG Wyse DG , et al The relationship between daily atrial tachyarrhythmia burden from implantable device diagnostics and stroke risk: the TRENDS study. Circ Arrhythm Electrophysiol 2009; 2: 474–480. 19843914 10.1161/CIRCEP.109.849638

[22] Saver JL Mattle HP Thaler D . Patent foramen ovale closure versus medical therapy for cryptogenic ischemic stroke: a topical review. Stroke 2018; 49: 1541–1548. 29760277 10.1161/STROKEAHA.117.018153

[23] Sanna T Diener HC Passman RS , et al Cryptogenic stroke and underlying atrial fibrillation. N Engl J Med 2014; 370: 2478–2486. 24963567 10.1056/NEJMoa1313600

[24] Gladstone DJ Spring M Dorian P , et al Atrial fibrillation in patients with cryptogenic stroke. N Engl J Med 2014; 370: 2467–2477. 24963566 10.1056/NEJMoa1311376

[25] Buck BH Hill MD Quinn FR , et al Effect of implantable vs prolonged external electrocardiographic monitoring on atrial fibrillation detection in patients with ischemic stroke: the PER DIEM randomized clinical trial. JAMA 2021; 325: 2160–2168. 34061146 10.1001/jama.2021.6128 PMC8170545

[26] Chen JZ Thijs VN . Presence of atrial fibrillation in stroke patients with patent foramen ovale: systematic review and meta-analysis. Front Neurol 2021; 12: 613758. 33935933 10.3389/fneur.2021.613758 PMC8081982

[27] Thijs VN Brachmann J Morillo CA , et al Predictors for atrial fibrillation detection after cryptogenic stroke: results from CRYSTAL AF. Neurology 2016; 86: 261–269. 26683642 10.1212/WNL.0000000000002282 PMC4733152

[28] Riordan M Opaskar A Yoruk A , et al Predictors of atrial fibrillation during long-term implantable cardiac monitoring following cryptogenic stroke. J Am Heart Assoc 2020; 9: e016040. 10.1161/JAHA.120.016040 PMC7792281 32689866

[29] Ziegler PD Rogers JD Ferreira SW , et al Long-term detection of atrial fibrillation with insertable cardiac monitors in a real-world cryptogenic stroke population. Int J Cardiol 2017; 244: 175–179. 28624331 10.1016/j.ijcard.2017.06.039

[30] Ziegler PD Rogers JD Ferreira SW , et al Real-world experience with insertable cardiac monitors to find atrial fibrillation in cryptogenic stroke. Cerebrovasc Dis 2015; 40: 175–181. 26314298 10.1159/000439063

[31] Wachter R Weber-Krüger M Seegers J , et al Age-dependent yield of screening for undetected atrial fibrillation in stroke patients: the Find-AF study. J Neurol 2013; 260: 2042–2045. 23632947 10.1007/s00415-013-6935-x PMC3734596

[32] Favilla CG Ingala E Jara J , et al Predictors of finding occult atrial fibrillation after cryptogenic stroke. Stroke 2015; 46: 1210–1215. 25851771 10.1161/STROKEAHA.114.007763

[33] Gladstone DJ Dorian P Spring M , et al Atrial premature beats predict atrial fibrillation in cryptogenic stroke: results from the EMBRACE trial. Stroke 2015; 46: 936–941. 25700289 10.1161/STROKEAHA.115.008714

[34] Sebasigari D Merkler A Guo Y , et al Biomarkers of atrial cardiopathy and atrial fibrillation detection on mobile outpatient continuous telemetry after embolic stroke of undetermined source. J Stroke Cerebrovasc Dis 2017; 26: 1249–1253. 28237125 10.1016/j.jstrokecerebrovasdis.2017.01.016

[35] Perlepe K Sirimarco G Strambo D , et al Left atrial diameter thresholds and new incident atrial fibrillation in embolic stroke of undetermined source. Eur J Intern Med 2020; 75: 30–34. 31952983 10.1016/j.ejim.2020.01.002

[36] Palà E Pagola J Juega J , et al B-type natriuretic peptide over N-terminal pro-brain natriuretic peptide to predict incident atrial fibrillation after cryptogenic stroke. Eur J Neurol 2021; 28: 540–547. 33043545 10.1111/ene.14579

[37] Bernstein RA Di Lazzaro V Rymer MM , et al Infarct topography and detection of atrial fibrillation in cryptogenic stroke: results from CRYSTAL AF. Cerebrovasc Dis 2015; 40: 91–96. 26182860 10.1159/000437018

[38] Campuzano O Perez-Serra A Iglesias A Brugada R . Genetic basis of atrial fibrillation. Genes Dis 2016; 3: 257–262. 30258896 10.1016/j.gendis.2016.09.003 PMC6150102

[39] Kitsiou A Sagris D Schäbitz WR Ntaios G . Validation of the AF-ESUS score to identify patients with embolic stroke of undetermined source and low risk of device-detected atrial fibrillation. Eur J Intern Med 2021; 89: 135–136. 33952425 10.1016/j.ejim.2021.04.003

[40] Kent DM Saver JL Kasner SE , et al Heterogeneity of treatment effects in an analysis of pooled individual patient data from randomized trials of device closure of patent foramen ovale after stroke. JAMA 2021; 326: 2277–2286. 34905030 10.1001/jama.2021.20956 PMC8672231

[41] Hindricks G Potpara T Dagres N , et al 2020 ESC guidelines for the diagnosis and management of atrial fibrillation developed in collaboration with the European Association for Cardio-Thoracic Surgery (EACTS): the task force for the diagnosis and management of atrial fibrillation of the European Society of Cardiology (ESC) developed with the special contribution of the European Heart Rhythm Association (EHRA) of the ESC. Eur Heart J 2020; 42: 373–498. 10.1093/eurheartj/ehaa612 32860505

[42] Tsivgoulis G Katsanos AH Grory BM , et al Prolonged cardiac rhythm monitoring and secondary stroke prevention in patients with cryptogenic cerebral ischemia. Stroke 2019; 50: 2175–2180. 31216964 10.1161/STROKEAHA.119.025169

[43] Svendsen JH Diederichsen SZ Højberg S , et al Implantable loop recorder detection of atrial fibrillation to prevent stroke (The LOOP Study): a randomised controlled trial. Lancet 2021; 398: 1507–1516. 34469766 10.1016/S0140-6736(21)01698-6

[44] Gaspardone A Sgueglia GA . Cryptogenic stroke over 60 years of age: should patent foramen ovale be closed? Eur Heart J Suppl 2020; 22: E82–E86. 10.1093/eurheartj/suaa067 PMC7270899 32523446

[45] Hart RG Sharma M Mundl H , et al Rivaroxaban for stroke prevention after embolic stroke of undetermined source. N Engl J Med 2018; 378: 2191–2201. 29766772 10.1056/NEJMoa1802686

[46] Diener HC Sacco RL Easton JD , et al Dabigatran for prevention of stroke after embolic stroke of undetermined source. N Engl J Med 2019; 380: 1906–1917. 31091372 10.1056/NEJMoa1813959

[47] Feil K Heinrich J Küpper C , et al Catch-up-ESUS—follow-up in embolic stroke of undetermined source (ESUS) in a prospective, open-label, observational study: study protocol and initial baseline data. BMJ Open 2019; 9: e031716. 10.1136/bmjopen-2019-031716 PMC6924738 31822542

