# Supplementary material for: Monitoring for atrial fibrillation prior to patent foramen ovale closure after cryptogenic stroke
Source: Int J Stroke. 2022 Sep 19;18(4):400–7. doi: 10.1177/17474930221124412 (PMC10037544; doi:10.1177/17474930221124412)
Supplement: sj-docx-1-wso-10.1177_17474930221124412 – Supplemental material for Monitoring for atrial fibrillation prior to patent foramen ovale closure after cryptogenic stroke [file sj-docx-1-wso-10.1177_17474930221124412.docx]

**ONLINE SUPPLEMENT**

Table S1: Current recommendations for cardiac rhythm monitoring in CS patients considered for PFO closure 2

Systematic literature search 3

Table S2: Overview of studies reporting on risk factors / predictors for detection of AF in CS patients 5

Table S3: Studies reporting on AF detection rates from various monitoring approaches 8

Table S4: Composed risk scores reported to be associated with the likelihood of AF detection 9

Table S5: PFO-Associated Stroke Causal Likelihood (PASCAL) classification system 10

References 11

# Table S1: Current recommendations for cardiac rhythm monitoring in CS patients considered for PFO closure

| Document | Criteria  (no recommendations for patients not meeting these criteria) | Recommended monitoring approach |
| --- | --- | --- |
| SCAI expert consensus statement^23^ | Age >40 years | Cardiac monitoring for approximately 4 weeks |
|  | Age ≤40 years | Cardiac monitoring for 1-2 weeks, unless there are other AF risk factors |
| ESO guideline^24^ | Adults with ischemic stroke or TIA of undetermined origin | Prolonged cardiac monitoring (>24 hours), out-patient cardiac rhythm monitoring, implantable devices for prolonged monitoring. |
| European position paper^25^ | Age ≥65 years  Age 55 – 64 years with ≥1 risk factor* for AF  Age <55 years at high risk of AF (≥1 major risk factor*) | ICM monitoring for ≥6 months  After treatment decision, monitoring may be extended to full duration of ICM life. |
| Canadian stroke best practice recommendations^26^ | No AF on initial short-term ECG monitoring, potential candidates for OAC. | Prolonged ECG monitoring for ≥2 weeks |
| Australian Clinical Guidelines for Stroke Management^27^ | Patients <60 years with a PFO considered the likely cause of stroke after exclusion of other etiologies. | 24hr Holter monitor, longer term monitoring could be considered if there is a high clinical suspicion for AF |
| AHA/ASA Guidelines^28^ | No contraindication to OAC | Long-term rhythm monitoring to detect intermittent AF |
| AAN Practice Advisory^29^ | At risk of AF | Prolonged cardiac monitoring for ≥28 days |

*Risk factors for AF: uncontrolled hypertension, structural heart alterations, uncontrolled diabetes, congestive heart failure, obesity, atrial runs, pulmonary disease, thyroid disease.

# Systematic literature search

A systematic literature search was conducted to identify literature reporting on risk factors or predictors for AF detection by systematic prolonged ECG monitoring in cryptogenic stroke patients. The results from this search were used to identify risk factors or predictors for the detection of AF that could be applied in a personalized approach, utilizing various monitoring modalities depending on the likelihood of detecting AF after a cryptogenic stroke.

This search was conducted according to the following search protocol.

Database: PubMed (https://pubmed.ncbi.nlm.nih.gov/)

Search string: ("cryptogenic stroke"[Title/Abstract] OR "embolic stroke of undetermined source"[Title/Abstract]) AND "atrial fibrillation"[Title/Abstract] AND ("detection"[Title/Abstract] OR "monitoring"[Title/Abstract])

Search period: Jan 1, 2014 - March 31, 2021

Filters: English language

Selection criteria:

- The study should report on individual risk factors / predictors (i.e., no composed risk scores) for the detection of AF in patients diagnosed with cryptogenic stroke.
- Reported risk factors / predictors should be based on AF detection by means of a systematic ECG monitoring approach (after ECG monitoring for the initial diagnosis of stroke etiology) utilizing at least one of the following:
  - Long-term ambulatory ECG monitoring
  - External event-triggered recording
  - Mobile Cardiac (Outpatient) Telemetry
  - Insertable cardiac monitor

For example, this excludes AF detection triggered by patient-reported symptoms, occasional / ad-hoc ECG recordings, etc.

- Studies including ≥100 patients with initial diagnosis of cryptogenic stroke.

Exclusion criteria:

- Articles not reporting on individual studies, i.e. meta-analyses, reviews, editorials, letters, comments, study design papers.
- Studies including mixed stroke populations, e.g., cryptogenic stroke patients and patients with stroke of known etiology.
- Studies on cardiac rhythm monitoring in other (non-cryptogenic stroke) populations or for other purposes than detection of AF.

The search in the PubMed database was conducted in April 2021 and returned 186 literature references, which were reviewed and selected according to the selection criteria listed above. See Figure S1 for a PRISMA flowchart. Assessment for eligibility eventually identified 22 articles with data from studies with ≥100 patients on risk factors or predictors for AF detection in cryptogenic stroke patients. Table S1 provides an overview of the studies that met the selection criteria and which were used as a first input for the manuscript.


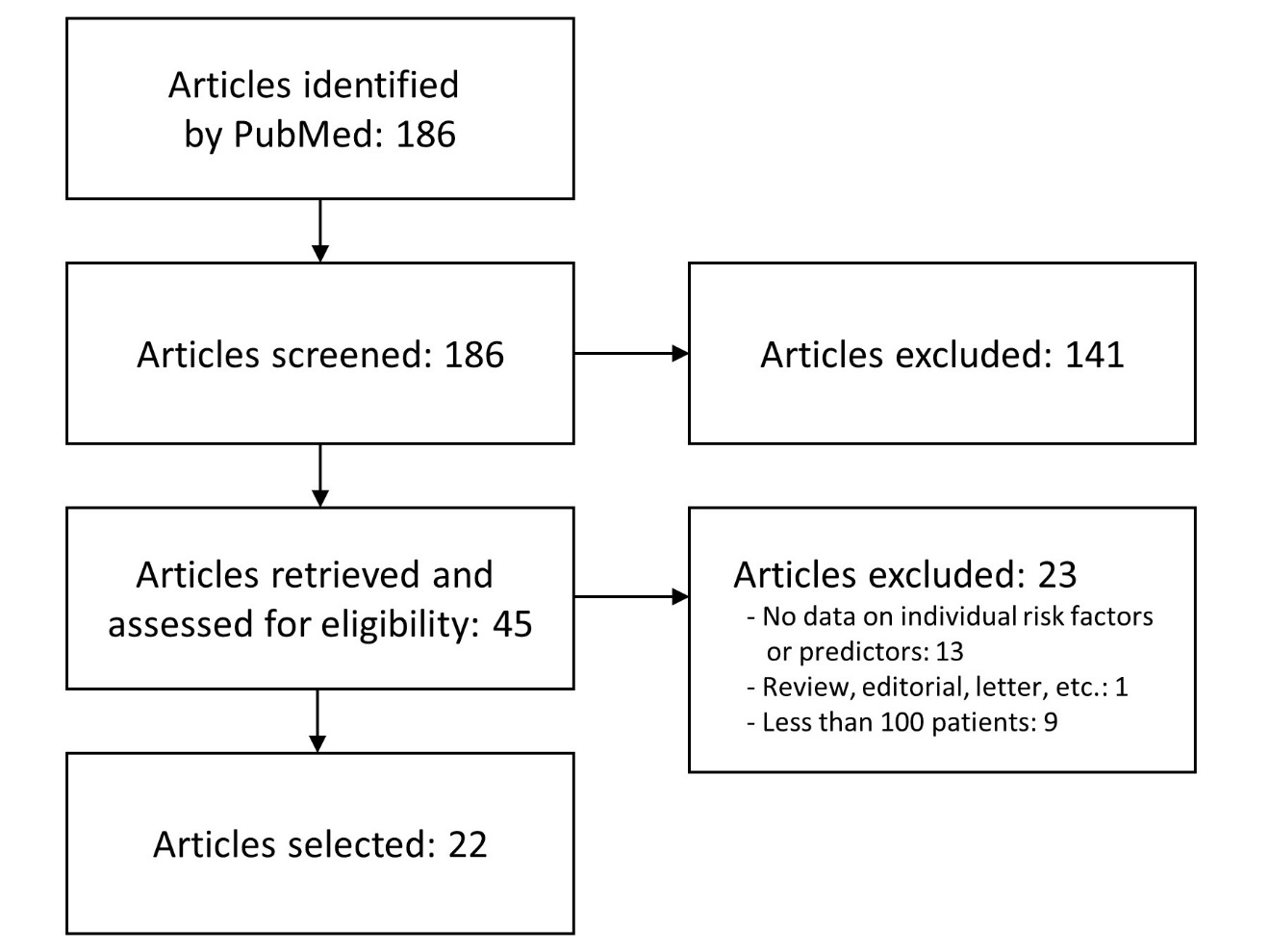


**Figure S1:** PRISMA flowchart of the systematic literature search to identify data on risk factors or predictors of AF detection in cryptogenic stroke patients. Initial screening involved a review of the title and abstract of the article and the type of the publication. A total of 141 articles were excluded by initial screening, for reasons including: Type of data reported (n=59, most frequently articles on AF detection but not reporting on risk factors or predictors of AF detection); Publication type (n=57, including reviews, meta-analyses, editorials, letters, study design papers); Studies including <100 patients (n=12); Articles not primarily related to cryptogenic stroke (n=10); Articles related to PFO closure with no specific data on AF detection (n=3).

# Table S2: Overview of studies reporting on risk factors / predictors for detection of AF in CS patients

| *Study* | *Patients with initial CS diagnosis* | *ECG monitoring* | *Risk factors/predictors for AF detection* |
| --- | --- | --- | --- |
| Acampa 2019A^1^  Acampa 2019B^2^ | N=222 | 7-day in-hospital ECG | Identified risk factors:   - Older age - High P-wave dispersion - Abnormal P-wave axis |
| Asaithambi 2018^3^ | N=234 | ICM >60 days | Identified risk factors:   - Older age - Female gender   LA enlargement or prolonged PR interval were not predictive of AF detection. |
| Bernstein 2015^4^ | N=212 | ICM | Identified risk factor:   - Coexisting chronic as well as acute brain infarcts   Acute brain infarction topography was not predictive for AF detection. |
| Carrazco 2017^5^ | N=100 | ICM | Identified risk factors:   - Older age - Increased PR interval - LA enlargement - Lower hemoglobin |
| Deferm 2021^6^ | N=191 | 30-day MCOT | Identified risk factors:  LA mechanics and particularly the LA booster pump function assessed early after CS. |
| Doijiri 2021^7^ | N=677 | 12-lead ECG  Continuous cardiac monitoring  24-hr Holter ECG | Spontaneous echo contrast was associated with AF detection ≤4 days after stroke.  Large infarctions predicted AF detection at >4 days. |
| Favilla 2015^8^ | N=227 | 28-day MCOT | Identified risk factors:   - Older age - Prior cortical or cerebellar infarction |
| Gladstone 2015^9^ | N=237 | 30-day event monitoring | Identified risk factors:   - Number of atrial premature beats on 24-hr Holter recording |
| Kneihsl 2019^10^ | N=143 | 24-hr Holter monitoring  In-hospital pulse controls (median: 15 days) | Increased NT-proBNP and to a lesser extent hypercoagulation markers are associated with AF-related stroke. Specifically NT-proBNP seems helpful in selecting CS patients for extended cardiac monitoring. |
| Makimoto 2017^11^ | N=146 | ICM | Patients with ESUS in the posterior cerebral artery territory had a higher rate of subclinical AT detection than those with other infarct localizations. |
| Marks 2020^12^ | N=178 | ICM | Older age, diastolic dysfunction, PACs, increased P-wave dispersion may help to predict higher risk of AF detection. P-wave dispersion was the only independent predictor. |
| Muscari 2020^13^ | N=191 | Continuous ECG monitoring | “ACTEL” score, composed of age ≥75 yrs, hypercholesterolemia, tricuspid regurgitation ≥ mild-to-moderate, LV end diastolic volume, LA ≥4 cm, allows identification of CS patients with AF. |
| Pagola 2020^14^ | N=264 | Continuous ECG monitoring within first 28 days | Detection of paroxysmal AF in patients with:   - Older age - Hemorrhagic infarction - Large vessel occlusion - Large LA volume index - Increased BNP   Age and large vessel occlusion independently associated with paroxysmal AF detection. |
| Pala 2021^15^ | N=218 | 28-days ECG monitoring | Both BNP and NT-proBNP were increased in patients with AF detection, but BNP achieves higher specificity. |
| Riordan 2020^16^ | N=293 | ICM | Older age is the most powerful predictor of occult AF in CS patients.  CHA_2_DS_2_-VASc score was also associated with higher risk of AF, but with a weaker association than age alone. |
| Rubio Campal 2019^17^ | N=100 | 21-day Holter monitoring | Patients with subclinical AF were older, more frequently had LA enlargement, renal impairment and slower mean heart rate.  Persistent bradycardia (≤60 bpm) was a powerful and significant risk factor for AF detection. |
| Sebasigari 2017^18^ | N=196 | Continuous heart rhythm monitoring | In unadjusted analysis, patients with AF were older and had larger LA diameter. In a multivariable model, age ≥60 years was the only predictor for AF. Atrial biomarkers were weakly associated with AF. |
| Sudacevschi 2016^19^ | N=171 | Long-duration rhythm cardiac Holter monitoring | Independent predictors for detection of paroxysmal AF:   - Age >70 years - PAC on ECG - LV hypertrophy on TEE - Previous white matter lesions on MRI |
| Thijs 2016^20^ | N=221 | ICM | Older age and prolonged PR interval were independently associated with increased AF detection rate, but only with moderate predictive value.  Independent predictors for AF detection at 12 months:   - Age: HR per decade = 1.9 [1.3–2.8], p= 0.0009 - PR interval: HR per 10 ms = 1.3 [1.2–1.4], p<0.0001 |
| Vollmuth 2019^21^ | N=104 | ICM | AF detection rate by ICM was not related to MRI imaging pattern (lesion size or volume, arterial vessel distribution, number of affected territories). |
| Xu 2020^22^ | N=389 | ICM | Older age and enlarged LA are predictors for LA detection. |

# Table S3: Studies reporting on AF detection rates from various monitoring approaches

| *Study* | *Monitoring approach / patients* | *AF detection* |
| --- | --- | --- |
| Bernstein 2021^30^  (STROKE-AF) | ICM monitoring vs. usual care (12-lead ECG, Holter monitoring, telemetry, event recording)  496 ischemic stroke patients (CS/ESUS excluded)  (67.1±9.4 years) | 12 months:  ICM: 12/1%  Usual care: 1.8% |
| Buck 2021^31^  (PER DIEM) | ICM monitoring vs. 4 weeks external loop recording  300 ischemic stroke patients (66% with stroke of undetermined etiology) | 12 months:  ICM: 15.3%  External loop recorder: 4.7% |
| Gladstone 2014^32^  (EMBRACE) | 30-day triggered event recording vs. 24-hour monitoring  280 patients with cryptogenic stroke or TIA (72.5±8.5 years, PFO prevalence not reported) | 30-day triggered event recording: 16.1%  24-hour monitoring: 3.2% |
| Kitsiou 2021^33^ | ICM monitoring  123 ESUS patients (65±9 years, 18% with PFO) | ≥36 months: 41.4% |
| Riordan 2020^16^ | ICM monitoring  293 CS patients (patients with AF: 72.1±10.8 years, patients without AF: 67.5±13.3 years, PFO prevalence not reported) | At mean follow-up of 22±12 months: 25%  36 months: 32% |
| Sanna 2014^34^  (CRYSTAL AF) | ICM vs. conventional follow-up (control)  441 CS patients  ICM: 61.6±11.4 years, 23.5% with PFO  Control: 61.4±11.3 years, 20.9 with PFO | 6 months:  ICM: 8.9%  Control: 1.4%  12 months:  ICM: 12.4%  Control: 2.0%  36 months:  ICM: 30.0%  Control: 3.0% |
| Svendsen 2021^35^  (LOOP) | ICM monitoring vs. usual care  6,004 patients with no AF and at least 1 of 4 conditions: hypertension, diabetes, prior stroke, heart failure (74.7±4.1 years, PFO prevalence not reported) | Median monitoring duration: 39.3 months  ICM: 31.8%  Usual care: 12.2% |
| Ziegler 2017^36^ | ICM monitoring  1,247 CS patients (65.3±13.0 years, PFO prevalence not reported) | 2 years: 21.5% |

# Table S4: Composed risk scores reported to be associated with the likelihood of AF detection

| *Risk score* | *Risk factors* | *Points* |
| --- | --- | --- |
| CHADS_2_  Gage et al.^37^ | Congestive Heart Failure  Hypertension (>140/90 mmHg)  Age ≥75 years  Diabetes Mellitus  Prior TIA or stroke | 1  1  1  1  2 |
| CHA_2_DS_2_VASc  Lip et al.^38^ | Congestive Heart Failure  Hypertension  Age ≥ 75 years  Diabetes  Stroke  Vascular disease  Age ≥ 65 years  Sex category, female | 1  1  2  1  2  1  1  1 |
| AF-ESUS  Ntaios et al.^39^ | Age ≥60 years  Hypertension  Left ventricular hypertrophy reported at echocardiography  Left atrial diameter >40 mm  Left ventricular ejection fraction <35%  Supraventricular extrasystole recorded during all available 12-lead standard electrocardiograms during hospitalization for ESUS  Subcortical infarct  Presence of non-stenotic carotid plaques | 3  2  -1  2  -3  1  -2  -3 |
| RoPE  Kent et al.^40^ | No history of:  Hypertension  Diabetes  Stroke or TIA  Non-smoker  Cortical infarct on imaging  Age (years)  18-29  30-39  40-49  50-59  60-69  >70 | 1  1  1  1  1  5  4  3  2  1  0 |
| PASCAL  Elgendy et al.^41^  Kent et al. ^42^ | See Table S4 |  |

# Table S5: PFO-Associated Stroke Causal Likelihood (PASCAL) classification system

| *Risk Grade* | *Features* | *Causal relatedness* | |
| --- | --- | --- | --- |
|  |  | *Low RoPE score* | *High RoPE score* |
| Very high risk | PFO + straddling thrombus | Definite | Definite |
| High risk | Both of:  1A. PFO + *or*  1B. Large shunt PFO  *AND*  2. PE or DVT preceding index infarct | Probable | Highly Probable |
| Medium risk | ANY of:  1. PFO + ASA  2. Large shunt PFO | Possible | Probable |
| Low risk | Small shunt PFO without ASA | Unlikely | Possible |

ASA: atrial septal aneurysm; DVT: deep vein thrombosis; PE: pulmonary embolism; PFO: patent foramen ovale; RoPE: Risk of Paradoxical Embolism (see Table S3).

High RoPE score: ≥7 points; Low RoPE score: <7 points.

# References

1. Acampa M, Lazzerini PE, Guideri F, et al. Electrocardiographic Predictors of Silent Atrial Fibrillation in Cryptogenic Stroke. *Heart Lung Circ* 2019; 28: 1664-1669.

2. Acampa M, Lazzerini PE, Guideri F, et al. P Wave Dispersion and Silent Atrial Fibrillation in Cryptogenic Stroke: The Pathogenic Role of Inflammation. *Cardiovasc Hematol Disord Drug Targets*

2019; 19: 249-252.

3. Asaithambi G, Monita JE, Annamalai MR, et al. Prevalence of atrial fibrillation with insertable cardiac monitors in cryptogenic stroke: A single-center experience. *J Electrocardiol* 2018; 51: 973-976.

4. Bernstein RA, Di Lazzaro V, Rymer MM, et al. Infarct Topography and Detection of Atrial Fibrillation in Cryptogenic Stroke: Results from CRYSTAL AF. *Cerebrovasc Dis* 2015; 40: 91-96.

5. Carrazco C, Golyan D, Kahen M, et al. Prevalence and Risk Factors for Paroxysmal Atrial Fibrillation and Flutter Detection after Cryptogenic Ischemic Stroke. *J Stroke Cerebrovasc* 2018; 27: 203-209.

6. Deferm S, Bertrand PB, Churchill TW, et al. Left Atrial Mechanics Assessed Early during Hospitalization for Cryptogenic Stroke Are Associated with Occult Atrial Fibrillation: A Speckle-Tracking Strain Echocardiography Study. *J Am Soc Echocardiog* 2021; 34: 156-165.

7. Doijiri R, Ueno Y, Kikuno M, et al. Different aspects of early and late development of atrial fibrillation during hospitalization in cryptogenic stroke. *Sci Rep* 2021; 11: 7127.

8. Favilla CG, Ingala E, Jara J, et al. Predictors of finding occult atrial fibrillation after cryptogenic stroke. *Stroke* 2015; 46: 1210-1215.

9. Gladstone DJ, Dorian P, Spring M, et al. Atrial premature beats predict atrial fibrillation in cryptogenic stroke: results from the EMBRACE trial. *Stroke* 2015; 46: 936-941.

10. Kneihsl M, Gattringer T, Bisping E, et al. Blood Biomarkers of Heart Failure and Hypercoagulation to Identify Atrial Fibrillation-Related Stroke. *Stroke* 2019; 50: 2223-2226.

11. Makimoto H, Kurt M, Gliem M, et al. High Incidence of Atrial Fibrillation After Embolic Stroke of Undetermined Source in Posterior Cerebral Artery Territory. *J Am Heart Assoc* 2017; 6: e007448.

12. Marks D, Ho R, Then R, et al. Real-world experience with implantable loop recorder monitoring to detect subclinical atrial fibrillation in patients with cryptogenic stroke: The value of p wave dispersion in predicting arrhythmia occurrence. *Int J Cardiol* 2021; 327: 86-92.

13. Muscari A, Barone P, Faccioli L, et al. Usefulness of the ACTEL Score to Predict Atrial Fibrillation in Patients with Cryptogenic Stroke. *Cardiology* 2020; 145: 168-177.

14. Pagola J, Juega J, Francisco-Pascual J, et al. Large vessel occlusion is independently associated with atrial fibrillation detection. *Eur J Neurol* 2020; 27: 1618-1624.

15. Palà E, Pagola J, Juega J, et al. B-type natriuretic peptide over N-terminal pro-brain natriuretic peptide to predict incident atrial fibrillation after cryptogenic stroke. *Eur J Neurol* 2021; 28: 540-547.

16. Riordan M, Opaskar A, Yoruk A, et al. Predictors of Atrial Fibrillation During Long-Term Implantable Cardiac Monitoring Following Cryptogenic Stroke. *J Am Heart Assoc* 2020; 9: e016040.

17. Rubio Campal JM, García Torres MA, Sánchez Borque P, et al. Detecting Atrial Fibrillation in Patients With an Embolic Stroke of Undetermined Source (from the DAF-ESUS registry). *Am J Cardiol* 2020; 125: 409-414.

18. Sebasigari D, Merkler A, Guo Y, et al. Biomarkers of Atrial Cardiopathy and Atrial Fibrillation Detection on Mobile Outpatient Continuous Telemetry After Embolic Stroke of Undetermined Source. *J Stroke Cerebrovasc Dis* 2017; 26: 1249-1253.

19. Sudacevschi V, Bertrand C, Chadenat ML, et al. Predictors of Occult Atrial Fibrillation in One Hundred Seventy-One Patients with Cryptogenic Transient Ischemic Attack and Minor Stroke. *J Stroke Cerebrovasc Dis* 2016; 25: 2673-2677.

20. Thijs VN, Brachmann J, Morillo CA, et al. Predictors for atrial fibrillation detection after cryptogenic stroke: Results from CRYSTAL AF. *Neurology* 2016; 86: 261-269.

21. Vollmuth C, Stoesser S, Neugebauer H, et al. MR-imaging pattern is not a predictor of occult atrial fibrillation in patients with cryptogenic stroke. *J Neurol* 2019; 266: 3058-3064.

22. Xu J, Sethi P, Biby S, et al. Predictors of atrial fibrillation detection and features of recurrent strokes in patients after cryptogenic stroke. *J Stroke Cerebrovasc Dis* 2020; 29: 104934.

23. Horlick E, Kavinsky CJ, Amin Z, et al. SCAI expert consensus statement on operator and institutional requirements for PFO closure for secondary prevention of paradoxical embolic stroke: The American Academy of Neurology affirms the value of this statement as an educational tool for neurologists. Catheter Cardiovasc Interv 2019; 93: 859-874.

24. Rubiera M, Aires A, Antonenko K, et al. European Stroke Organisation (ESO) guideline on screening for subclinical atrial fibrillation after stroke or transient ischaemic attack of undetermined origin. Eur Stroke J Epub ahead of print June 3, 2022. DOI: 10.1177/23969873221099478.

25. Pristipino C, Sievert H, D'Ascenzo F, et al. European position paper on the management of patients with patent foramen ovale. General approach and left circulation thromboembolism. EuroIntervention 2019; 14: 1389-1402.

26. Wein T, Lindsay MP, Cote R, et al. Canadian stroke best practice recommendations: Secondary prevention of stroke, sixth edition practice guidelines, update 2017. Int J Stroke 2018; 13: 420-443.

27. Australian Clinical Guidelines for Stroke Management - Chapter 4 of 8: Secondary Prevention, https://app.magicapp.org/#/guideline/8L0RME/section/EgV9pn (2021, accessed August 17 2021).

28. Kleindorfer DO, Towfighi A, Chaturvedi S, et al. 2021 Guideline for the Prevention of Stroke in Patients With Stroke and Transient Ischemic Attack: A Guideline From the American Heart Association/American Stroke Association. Stroke 2021; 52: e364-e467.

29. Messé SR, Gronseth GS, Kent DM, et al. Practice advisory update summary: Patent foramen ovale and secondary stroke prevention: Report of the Guideline Subcommittee of the American Academy of Neurology. Neurology 2020; 94: 876-885.

30. Bernstein RA, Kamel H, Granger CB, et al. Effect of Long-term Continuous Cardiac Monitoring vs Usual Care on Detection of Atrial Fibrillation in Patients With Stroke Attributed to Large- or Small-Vessel Disease - The STROKE-AF Randomized Clinical Trial. *JAMA* 2021;325:2169-2177.

31. Buck BH, Hill MD, Quinn FR, et al. Effect of Implantable vs Prolonged External Electrocardiographic Monitoring on Atrial Fibrillation Detection in Patients With Ischemic Stroke: The PER DIEM Randomized Clinical Trial. *JAMA* 2021; 325: 2160-2168.

32. Gladstone DJ, Spring M, Dorian P, et al. Atrial fibrillation in patients with cryptogenic stroke. *N Engl J Med* 2014; 370: 2467-2477.

33. Kitsiou A, Rogalewski A, Kalyani M, et al. Atrial Fibrillation in Patients with Embolic Stroke of Undetermined Source during 3 Years of Prolonged Monitoring with an Implantable Loop Recorder. *Thromb Haemostasis* 2021; 121: 826-833.

34. Sanna T, Diener H-C, Passman RS, et al. Cryptogenic stroke and underlying atrial fibrillation. N Engl J Med 2014; 370: 2478-2486.

35. Svendsen JH, Diederichsen SZ, Højberg S, et al. Implantable loop recorder detection of atrial fibrillation to prevent stroke (The LOOP Study): a randomised controlled trial. *Lancet* 2021; 398: 1507-1516.

36. Ziegler PD, Rogers JD, Ferreira SW, et al. Long-term detection of atrial fibrillation with insertable cardiac monitors in a real-world cryptogenic stroke population. *Int J Cardiol* 2017;244:175-179.

37. Gage BF, Waterman AD, Shannon W et al. Validation of clinical classification schemes for predicting stroke: results from the National Registry of Atrial Fibrillation. *JAMA* 2001;285:2864-2870.

38. Lip GYH, Frison L, Halperin JLH, Lane DA. Identifying patients at high risk for stroke despite anticoagulation: a comparison of contemporary stroke risk stratification schemes in an anticoagulated atrial fibrillation cohort. *Stroke* 2010;41:2731-2738

39. Ntaios G, Perlepe K, Lambrou D, et al. Identification of patients with embolic stroke of undetermined source and low risk of new incident atrial fibrillation: The AF-ESUS score. *Int J Stroke* 2021; 16: 29-38.

40. Kent DM, Ruthazer R, Weimar C, et al. An index to identify stroke-related vs incidental patent foramen ovale in cryptogenic stroke. *Neurology* 2013;81:619-625.

41. Elgendy AY, Saver JL, Amin Z, et al. Proposal for Updated Nomenclature and Classification of Potential Causative Mechanism in Patent Foramen Ovale-Associated Stroke. JAMA Neurol 2020; 77: 878-886.

42. Kent DM, Saver JL, Kasner SE, et al. Heterogeneity of treatment effects in an analysis of pooled individual patient data from randomized trials of device closure of patent foramen ovale after stroke. *JAMA* 2021;326:2277-2286.
